# Bacterial neurotoxins: The snake venoms of the microbial world—a clinical and bioengineering approach

**DOI:** 10.1016/j.crmicr.2026.100651

**Published:** 2026-07-24

**Authors:** Hanieh Banaeyoun, Yaser Solhi Digehsara, Behrouz Golichenari

**Affiliations:** a Department of Biotechnology, Faculty of New Sciences and Technologies, Semnan University, Semnan, Iran; b Cellular and Molecular Research Center, School of Medicine, Guilan University of Medical Sciences, Rasht, Iran

**Keywords:** Neurotoxin, Signaling, Nervous system, Snare, Toxin, GPCR

## Abstract

The central and peripheral nervous systems constitute highly organized and dynamic networks that rely on precise regulation of synaptic transmission, membrane integrity, cytoskeletal stability, and intracellular signaling. Disruption of these tightly controlled processes can lead to a wide range of neurological disorders. Among the various modulators of neural function, bacterial neurotoxins represent a unique class of exotoxins that selectively target key neuronal components with remarkable molecular specificity. Traditionally studied in the context of infectious pathogenesis, bacterial neurotoxins are now increasingly recognized as powerful molecular probes of synaptic machinery and intracellular signaling pathways. In addition to their direct effects on neurons, accumulating evidence indicates that bacterial neurotoxins also participate in neuroimmune interactions, contribute to neuroinflammatory cascades, and may impact gut-brain axis signaling and long-term neurodegenerative vulnerability.

Advances in structural biology and protein engineering have shifted the field from descriptive toxicology to translational neurobiology. Engineered toxin derivatives and attenuated catalytic variants are being investigated as tools for neural circuit modulation, targeted drug delivery, and the treatment of movement disorders, chronic pain, and other neurological conditions. This review offers a comprehensive and critical synthesis of bacterial neurotoxins, integrating molecular mechanisms of action with their pathological consequences, emerging research and therapeutic applications. By combining insights from infectious disease biology and systems neuroscience, we seek to outline both the opportunities and the unresolved challenges that shape the future direction of bacterial neurotoxin research in precision neurology.

## Highlights

• This review provides a mechanistic classification of bacterial neurotoxins.
• Neurotoxins are discussed as modulators of neural and neuroimmune networks.
• Emerging links between bacterial neurotoxins and the gut–brain axis are highlighted.
• The role of neurotoxins in neurodegenerative disorders is critically evaluated.
• Engineered neurotoxin platforms show promise for targeted neural therapeutics.
• Current translational opportunities and limitations in precision neurology are reviewed.

## Graphical abstract

**Figure:**
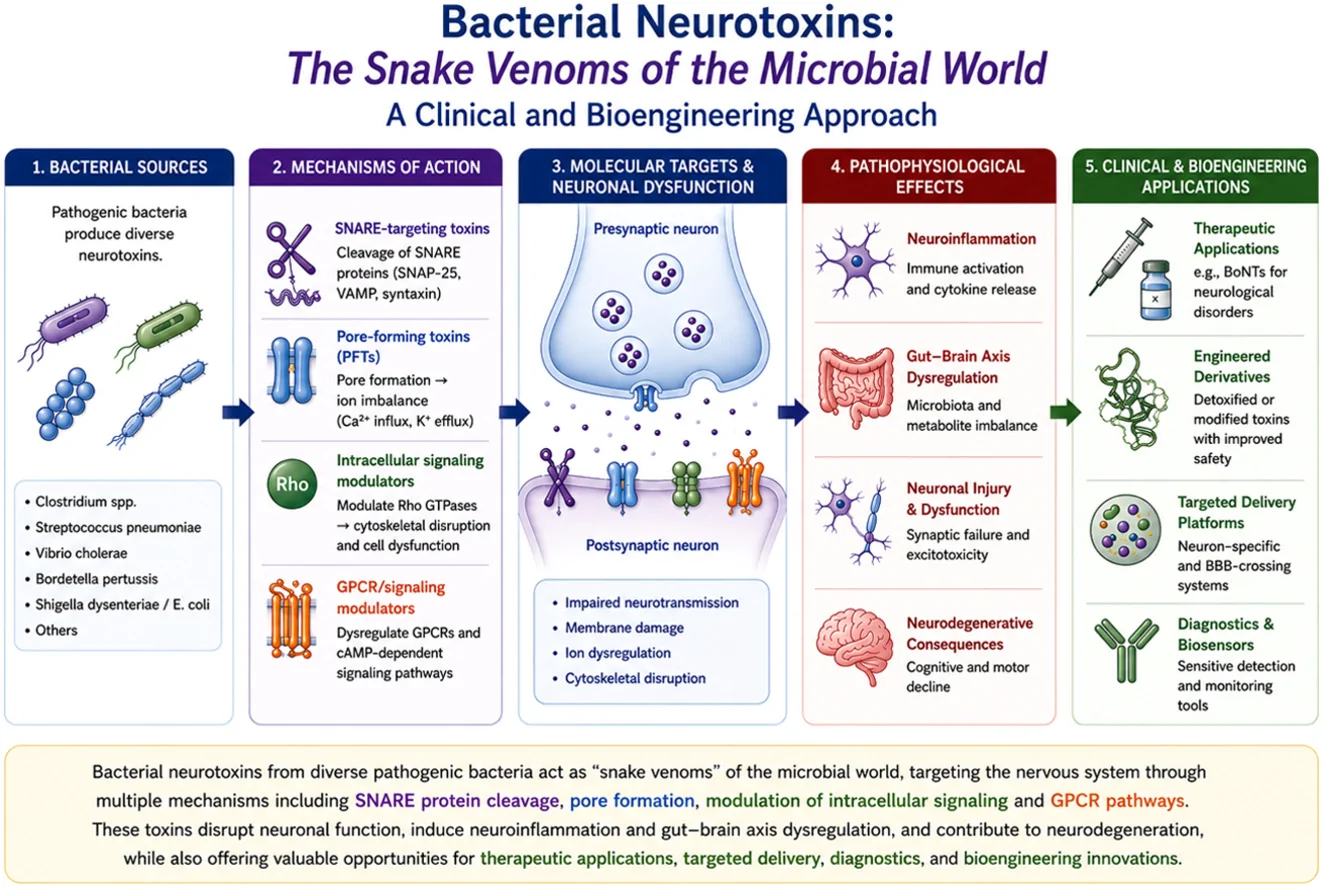

## 1 Introduction

The central and peripheral nervous systems constitute a highly organized, dynamic, and complex network of neurons and glial cells that coordinate electrical and chemical signaling to enable information processing, regulation of physiological responses, and maintenance of systemic homeostasis (Kandel et al., 2000). The integrity of this system depends on the preservation of membrane architecture, cytoskeletal stability, tightly regulated synaptic transmission, and finely tuned intracellular signaling cascades (Schmick and Bastiaens, 2014; Pfaff et al., 2022). Disruption at either the molecular or network level may lead to a broad spectrum of neurological disorders, ranging from acute neuromuscular paralysis and synaptopathies to progressive neurodegenerative conditions, including motor neuron degeneration and cognitive impairment (Goedert and Spillantini, 2006; Taylor, Brown Jr and Cleveland, 2016).

Among the diverse factors capable of perturbing neural function, infectious agents occupy a distinctive position. Pathogenic bacteria mediate their pathogenic effects not only through direct tissue invasion but also through the secretion of highly specific exotoxins that target and reprogram essential host cellular pathways (Rasko and Sperandio, 2010; Aktories et al., 2011). These toxins alter the intracellular environment by mechanisms such as inhibition of protein synthesis, cytoskeletal disruption, modulation of membrane permeability, and interference with vesicular trafficking, thereby facilitating bacterial survival and disease progression (Popoff and Poulain, 2010).

Bacterial neurotoxins represent a specialized subset of these exotoxins that selectively target critical neuronal structures and processes. These molecules typically bind to neuronal receptors with high specificity, undergo internalization, and interfere with essential components of synaptic transmission. The consequences include impaired neurotransmitter release, altered neuronal excitability, or functional synaptic paralysis. Initially regarded solely as virulence factors, these molecules exhibit remarkable specificity that reflects highly evolved host-pathogen molecular interactions (Popoff and Poulain, 2010; Pirazzini et al., 2017).

For decades, research on bacterial neurotoxins was largely confined to the context of infectious disease and clinical manifestations, focusing on mechanisms of toxicity, prevention, and neutralization (Masuyer et al., 2014). However, advances in molecular biology, structural biology, and genetic engineering have enabled precise identification of their molecular targets and signaling pathways. These discoveries have demonstrated that their specificity arises from highly selective, structure-dependent interactions with key neuronal components, rendering them valuable tools for targeted modulation of neural function (Pirazzini et al., 2017; Dong, Masuyer and Stenmark, 2019).

Consequently, bacterial neurotoxins have evolved from being considered exclusively destructive agents to becoming powerful research tools in neuroscience. They have facilitated detailed investigation of vesicular release mechanisms, synaptic protein organization, and neural network dynamics. (Jahn and Fasshauer, 2012). Furthermore, modification or removal of catalytic domains while preserving neuronal targeting properties has enabled the development of engineered derivatives with therapeutic potential. (Masuyer et al., 2014). Such strategies are currently being explored for the treatment of movement disorders, chronic pain syndromes, dystonia, and other neurological conditions (Dressler and Benecke, 2007).

Despite substantial advances in understanding their structure and function, integration of pathogenic insights with translational and research applications remains incomplete. Critical questions persist regarding safety limitations, long-term functional stability, targeting specificity, and potential consequences of therapeutic use (Simpson, 1981). A comprehensive and critical synthesis of current knowledge is therefore necessary to clarify both opportunities and limitations in this field.

Recent advances in molecular neurobiology and structural research have reshaped the understanding of bacterial neurotoxins, positioning them not only as agents of neural injury but also as precise modulators of neuronal function. Integrating their molecular mechanisms, pathological effects, and emerging translational relevance offers a broader perspective on how these specialized microbial factors intersect with neural organization, dysfunction, and therapeutic innovation (Rossetto and Montecucco, 2008; Foster, 2009).

Rather than considering bacterial neurotoxins solely as virulence factors, this review adopts the perspective that their highly evolved molecular precision can provide important insights into neuronal biology while offering a foundation for the development of next-generation neurotherapeutic and bioengineering strategies.

## 2 Bacterial neurotoxins

Bacterial neurotoxins comprise a heterogeneous group of protein exotoxins produced by pathogenic bacteria that impair nervous system function through distinct molecular mechanisms. Among them, botulinum neurotoxins (BoNTs) and tetanus neurotoxin (TeNT) are regarded as the prototypical bacterial neurotoxins because of their highly specific neuronal tropism and receptor-mediated internalization. Several other bacterial toxins with neuroactive properties produce neurological effects through disruption of membrane integrity, intracellular signaling pathways, neurovascular homeostasis, or neuroimmune response (Popoff and Poulain, 2010; Ribet and Cossart, 2010; Aktories, 2011). Depending on their molecular targets, these toxins affect both the central and peripheral nervous systems by inhibiting neurotransmitter release, altering neuronal excitability, inducing paralysis, or modulating neural network activity (Rossetto and Montecucco, 2008).

**Fig. 1:**
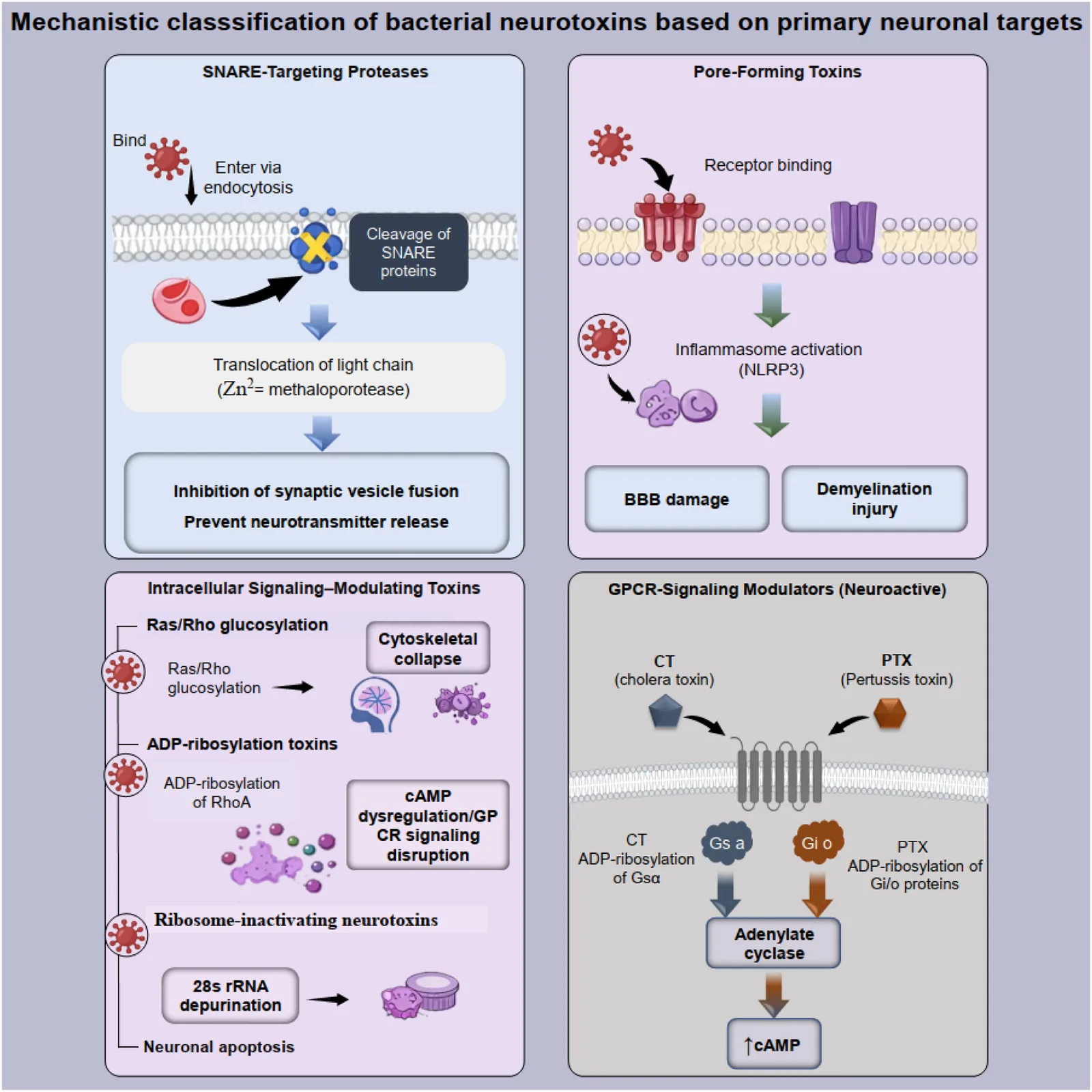
Mechanistic classification of bacterial neurotoxins based on primary neuronal targets. Bacterial neurotoxins are functionally categorized into four major classes: SNARE-targeting proteases, pore-forming toxins (PFTs), intracellular signaling–modulating toxins, and neuroactive GPCR-signaling modulators. Each class disrupts neuronal function through distinct molecular mechanisms that affect synaptic transmission, membrane integrity, intracellular signaling, or second messenger pathways (Adapted from Voth and Ballard, 2005; Sánchez and Holmgren, 2008; Locht, Coutte and Mielcarek, 2011; Rossetto, Pirazzini and Montecucco, 2014; Peraro and Van Der Goot, 2016).

The functional classification of bacterial neurotoxins is based on their molecular mechanisms of action, cellular targets, and neurobiological consequences. This approach allows for a comparison of pathogenic, regulatory, and therapeutic effects. Accordingly, bacterial neurotoxins can be grouped into four principal mechanistic categories: (i) Targeting the synaptic vesicle fusion machinery (SNARE complex), (ii) pore-formation and disruption of membrane homeostasis, (iii) modulation of intracellular signaling pathways, or (iv) interference with G-protein-coupled receptor (GPCR) signaling through ADP-ribosylation of heterotrimeric G proteins (Simon, Aktories and Barbieri, 2014; Kumar et al., 2019). Fig. 1 summarizes the mechanistic classification of bacterial neurotoxins based on their neuronal targets.

### 2.1 SNARE-targeting neurotoxins

#### 2.1.1 Structure and molecular mechanism of action on the snare complex

The most prominent bacterial neurotoxins targeting the synaptic vesicle fusion machinery are the clostridial neurotoxins, which include botulinum neurotoxins (BoNTs) and tetanus neurotoxin (TeNT) (Schiavo, Matteoli and Montecucco, 2000; Rossetto, Pirazzini and Montecucco, 2014). These protein toxins are produced by anaerobic Gram-positive spore-forming bacteria of the genus *Clostridium* and belong to the classical A–B toxin family. They inhibit neurotransmitter release through highly specific cleavage of SNARE proteins (Popoff, 2026). Their genetic diversity extends beyond the classical serotypes A-G and includes multiple subtypes and mosaic variants, reflecting substantial evolutionary and functional complexity (Hill and Smith, 2012).

These neurotoxins are synthesized as approximately 150 kDa single-chain polypeptides that undergo proteolytic activation to generate a dichain structure composed of a light chain (LC) with catalytic activity and a heavy chain (HC) responsible for binding and translocation. The two chains remain linked via a disulfide bond. The heavy chain consists of two functional domains: the C-terminal binding domain (H_C), which mediates recognition of neuronal receptors, and the N-terminal translocation domain (H_N), which facilitates LC delivery from endosomes into the cytosol (Monash et al., 2025).

Neuronal binding follows a dual-receptor model. Initially, the interaction between H_C and polysialogangliosides on the presynaptic membrane concentrates the toxin at the neuronal surface (Rummel et al., 2007). Subsequently, high-affinity binding to protein receptors, such as synaptotagmin I/II or synaptic vesicle protein 2 (SV2), induces receptor-mediated endocytosis. Serotype-specific receptor usage contributes to target selectivity (Dong et al., 2006; Jahn, Cafisoand Tamm, 2024).

Following endocytosis, endosomal acidification triggers conformational rearrangements within the H_N domain, promoting membrane insertion and translocation of the LC into the cytosol. Concurrent reduction of the interchain disulfide bond by cellular thioredoxin systems releases the active LC (Pirazzini et al., 2014). This pH-dependent step represents a critical regulatory checkpoint in toxin efficiency.

Within the cytosol, the LC functions as a zinc-dependent metalloprotease that cleaves SNARE proteins, such as synaptobrevin (VAMP), syntaxin, or SNAP-25, in a serotype-specific manner (Blasi et al., 1993; Schiavo et al., 1997). For example, BoNT/A and BoNT/E cleave SNAP-25 at distinct sites, whereas TeNT and several other serotypes preferentially target VAMP (Sikorra et al., 2008). These cleavages prevent the formation of the ternary SNARE complex required for synaptic vesicle fusion (Südhof, 2013). Notably, cleavage of a relatively small fraction of SNARE proteins is sufficient to completely block neurotransmitter release, underscoring the extraordinary catalytic efficiency of these toxins (Pantano and Montecucco, 2014). The prolonged duration of action of certain serotypes, particularly BoNT/A, is attributed to the extended intracellular half-life of the LC (Pellett et al., 2018).

#### 2.1.2 Functional differences and physiological consequences

Despite structural similarities and shared molecular mechanisms, differences in axonal transport and neuronal targeting account for distinct clinical manifestations (Skryabina et al., 2023). BoNTs generally remain at the neuromuscular junction, where inhibition of acetylcholine release leads to flaccid paralysis (Pirazzini et al., 2014). In contrast, TeNT undergoes retrograde axonal transport to the central nervous system and selectively targets inhibitory GABAergic and glycinergic interneurons (Popoff, 2026). Blockade of inhibitory neurotransmitter release disrupts the excitation-inhibition balance, resulting in sustained muscle spasms and spastic paralysis (Hassel, 2013).

Clinically, these properties have enabled widespread therapeutic application of BoNTs in neuromuscular disorders, spasticity, dystonia, chronic migraine, and selected pain syndromes (Dressler, 2012), whereas TeNT remains primarily relevant in the context of tetanus pathogenesis. Nevertheless, structural and functional studies of TeNT have substantially contributed to the fundamental understanding of synaptic transmission mechanisms (Zhou et al., 2022).

### 2.2 Pore-forming toxins and disruption of neuronal membrane homeostasis

In contrast to SNARE-targeting neurotoxins that act through precise enzymatic interference with synaptic machinery, another class of bacterial toxins exerts neurotoxic effects by directly compromising plasma membrane integrity (Peraro and Van Der Goot, 2016). Known as pore-forming toxins (PFTs), these molecules assemble transmembrane pores within lipid bilayers, thereby disrupting ionic and electrochemical homeostasis (Rojko and Anderluh, 2015). Although many PFTs are not strictly neuron-specific, they can induce significant direct or secondary neurotoxicity in the context of central nervous system infection or blood-brain barrier (BBB) compromise (Los et al., 2013).

Structurally, PFTs are classified into α- and β-pore-forming toxins. In neurological pathogenesis, cholesterol-dependent cytolysins (CDCs), a subset of β-PFTs, are particularly relevant (Gilbert, 2010; Ulhuq and Mariano, 2022). Pneumolysin produced by *Streptococcus pneumoniae*, and epsilon toxin from *Clostridium perfringens* represent prominent examples associated with neural injury (Finnie and Uzal, 2022; Narcisoet al., 2025).

#### 2.2.1 Molecular mechanism of pore formation

PFT activity progresses through sequential steps: membrane binding, surface oligomerization, prepore complex formation, and membrane insertion (Hu, Liu and Sun, 2021). Pneumolysin requires cholesterol binding for cytolytic activity (Lata et al., 2022). After membrane association, toxin monomers oligomerize into large ring-shaped complexes that undergo substantial β-structural rearrangements and insert into the lipid bilayer, forming pores several nanometers in diameter (Li et al., 2021).

At high concentrations, pore formation induces direct osmotic lysis. At sublytic levels, however, microperforations cause more subtle yet pathophysiologically significant effects, including dysregulated calcium signaling, activation of inflammatory pathways, and disruption of BBB integrity (Los et al., 2013; Sathyanarayana, Visweswariah and Ayappa, 2020).

Epsilon toxin, after proteolytic activation, binds to specific cellular receptors. Recent studies implicate the myelin and lymphocyte protein (MAL) as a potential receptor or co-receptor in oligodendrocytes, possibly explaining the heightened vulnerability of neural tissue (Dorca-Arévalo et al., 2024). Following oligomerization, stable multimeric pores form, exerting pronounced effects on brain endothelial cells and oligodendrocytes (Xin and Wang, 2019).

#### 2.2.2 Ionic dysregulation and neurotoxic consequences

Membrane pore formation allows for rapid influx of Ca²⁺ and efflux of K⁺ (González-Juarbe et al., 2017). Increased intracellular calcium activates calpains, phospholipases, and mitochondrial-dependent pathways that can lead to apoptosis or necrosis. Additionally, potassium efflux plays a crucial role in triggering NLRP3 inflammasome activation, which enhances local inflammatory responses (Muñoz-Planillo et al., 2013).

In pneumococcal meningitis, pneumolysin disrupts endothelial tight junctions and compromises BBB integrity, contributing to cerebral edema and worsening neuroinflammation (Petrović, no date). Epsilon toxin-induced BBB permeability and widespread neuronal depolarization are linked to abnormal glutamate release (Popoff and Poulain, 2010). Overactivation of NMDA receptors intensifies calcium-dependent excitotoxic cascades, potentially leading to seizures and rapid neuronal death (Wu et al., 2025).

In summary, neurotoxicity caused by PFTs results from the combined effects of ionic imbalance, inflammatory activation, and vascular injury, distinguishing them mechanistically from enzymatic SNARE-targeting neurotoxins (Bouillot, Reboud and Huber, 2018).

### 2.3 Toxins modulating intracellular neuronal signaling pathways

Beyond vesicle fusion inhibitors and pore-forming toxins, a third category of bacterial toxins directly reprograms intracellular signaling networks. Unlike SNARE-targeting toxins that enzymatically disable exocytotic machinery, or PFTs that disrupt membrane integrity, these toxins covalently modify key regulatory proteins, thereby altering actin cytoskeleton organization, dendritic dynamics, vesicular trafficking, and neuronal survival pathways. The resulting cellular reprogramming may impair synaptic efficiency, compromise plasticity, and, under severe conditions, induce neuronal death (Aktories and Barbieri, 2005).

Representative members include the Large Clostridial Toxins (LCTs), such as toxins A and B from *Clostridioides difficile*, lethal and hemorrhagic toxins from *Paeniclostridium sordellii*, alpha toxin from *Clostridium novyi*, and TpeL from *Clostridium perfringens* (Orrell and Melnyk, 2021). Although primarily implicated in extra-neural pathology, experimental evidence indicates that under certain conditions, they may exert neurotropic or neurotoxic effects (Popoff and Poulain, 2010).

#### 2.3.1 Molecular inactivation of small GTPases and downstream consequences

LCTs have a multidomain organization consisting of a receptor-binding domain, a translocation domain, and an N-terminal catalytic glucosyltransferase domain. Following receptor-mediated endocytosis, endosomal acidification enables the cytosolic delivery of the catalytic domain (Orrell and Melnyk, 2021).

In the cytosol, this domain uses UDP-glucose or UDP–N-acetylglucosamine to glucosylate crucial residues within the switch I/II regions of RhoA, Rac1, and Cdc42. This covalent modification disrupts GDP/GTP cycling and stabilizes these GTPases in an inactive state (Just, Selzer, et al., 1995; Genth et al., 2006). Given the central role of the Rho family in actin polymerization, dendritic spine formation, and synaptic active zone organization, their inactivation leads to actin cytoskeleton collapse and impaired synaptic vesicle priming (Aktories, 2011; Kalpachidou et al., 2019).

Similarly, C3 exoenzyme from *Clostridium botulinum* selectively ADP-ribosylates RhoA, disrupting actomyosin contractility and synaptic stability (Aktories and Hall, 1989).

In addition to structural effects, inhibition of Rac1 and Cdc42 suppresses pro-survival PI3K/Akt and MAPK pathways (Lamarche et al., 1996). Reduced survival signaling may activate stress-responsive transcription factors, upregulate pro-apoptotic genes, and trigger caspase-3 activation (Coleman and Olson, 2002). In neuronal models, these processes are linked to dendritic retraction, chromatin condensation, and apoptosis (Newey et al., 2005).

Furthermore, Shiga toxin type 2 produced by *Shigella dysenteriae* and certain pathogenic strains of *Escherichia coli*, has been recognized for its neurotoxic potential despite being primarily classified as a ribosome-inactivating toxin(Silva et al., 2017). Although its primary mechanism involves ribosomal inhibition, it can also disrupt neuronal survival signaling through the activation of endoplasmic reticulum stress and inflammatory pathways (Johannes and Römer, 2010; Park et al., 2017). Binding to the glycolipid receptor Gb3 facilitates cellular entry (Chan and Ng, 2016), and clinical cases have linked exposure to encephalopathy and seizures (Joseph et al., 2020).

In summary, signaling-modulating toxins, by fundamentally reprogramming intracellular regulatory networks, may have more sustained and potentially degenerative effects compared to acute SNARE-targeting neurotoxins.

### 2.4 GPCR-signaling modulators (Neuroactive)

Distinct from toxins that compromise membrane integrity or disable cytoskeletal and vesicular regulatory proteins, another class of bacterial toxins exerts its effects by rewiring G-protein–coupled receptor (GPCR) signaling within neuronal and neuroimmune circuits (Foster, Kinney and Iglewski, 1984; Wilson and Ho, 2010). These toxins do not rely on pore formation or structural disruption of small GTPases; instead, they selectively modify heterotrimeric G-protein α-subunits, thereby perturbing the homeostasis of cyclic nucleotide signaling, altering neuromodulatory receptor responsiveness, and reshaping communication between neurons, glia, and immune cells (Woolkalis, 2020). The resulting imbalance in intracellular second-messenger dynamics can alter synaptic efficacy, disturb neurotransmitter release patterns, and, under conditions of sustained exposure, drive maladaptive inflammatory or excitability states (Neves, Ram and Iyengar, 2002).

Representative neuroactive members of this group include cholera toxin (CT) produced by *Vibrio cholerae* and pertussis toxin (PTX) produced by *Bordetella pertussis* (Vanden Broeck, Horvath and De Wolf, 2007; Carbonetti, 2010). Although historically associated with epithelial or respiratory disease, mounting experimental evidence indicates that both toxins exert profound effects on enteric, sensory, and autonomic neurons due to their capacity to manipulate GPCR-dependent intracellular signaling programs (Popoff, 2024).

#### 2.4.1 ADP-ribosylation of G-protein subunits and disruption of cAMP homeostasis

Both CT and PTX share a structurally conserved AB-type architecture comprising a catalytic A subunit and a receptor-binding B oligomer. Following receptor-mediated endocytosis and endosomal trafficking, the A subunit undergoes conformational rearrangements that enable its translocation into the cytosol, where it catalyzes NAD⁺-dependent ADP-ribosylation of defined G-protein α-subunits (Sandvig and Van Deurs, 2002).

Cholera toxin binds with high affinity to GM1 gangliosides, which are abundantly expressed on both intestinal epithelial cells and enteric neurons. After retrograde trafficking to the endoplasmic reticulum and reductive activation, the A1 fragment modifies Gsα, locking it in a constitutively active GTP-bound state. This persistent activation leads to uncontrolled stimulation of adenylate cyclase and sustained intracellular cAMP accumulation (Haan and Hirst, 2004). In neuronal systems, elevated cAMP enhances PKA and EPAC signaling, disrupts Ca²⁺-channel regulation, modifies vesicular release probability, and promotes hyperexcitability within secretomotor circuits of the enteric nervous system (Bos, 2006). These alterations collectively account for CT-induced enteric neuronal dysregulation, which extends beyond epithelial pathology to involve neurogenic components of fluid secretion and motility (Jiang et al., 1993).

In contrast, pertussis toxin targets the Gi/o family of inhibitory G proteins. After binding to complex N-linked glycans and undergoing proteolytic activation within endosomes, its enzymatic S1 subunit ADP-ribosylates Gi/oα, preventing proper coupling between GPCRs and their cognate G proteins. This functional decoupling renders Gi/o inactive, thereby removing inhibitory control over adenylate cyclase and again resulting in elevated cytosolic cAMP (Tang et al., 2026). In neuronal and immune cells, this loss of Gi/o signaling disrupts chemokine receptor function, alters Gβγ-dependent ion-channel modulation, impairs synaptic inhibition, and dysregulates neuroimmune communication (Mangmool and Kurose, 2011; Shouman and Benarroch, 2021). These changes underpin PTX-associated phenotypes such as altered chemokine signaling, aberrant immune–neuron interactions, and destabilized autonomic reflex circuits (Zhao et al., 2025).

Collectively, GPCR-signaling modulators induce neurotoxicity through biochemical, rather than structural, perturbations. By reprogramming the intracellular logic of G-protein–mediated signaling, they generate sustained disturbances in cAMP-dependent pathways, modify neuronal excitability, and shape neuroimmune responses in ways that differ fundamentally from the acute cytopathic mechanisms of pore-forming or SNARE-targeting neurotoxins (Cheng et al., 2023; Yadav and Zaccolo, 2025). Their actions highlight the vulnerability of neuronal networks to subtle enzymatic modifications of receptor-proximal signaling machinery and underscore the diversity of microbial strategies for manipulating neural function.

A comparative overview of the major bacterial neurotoxins, their molecular mechanisms, neuronal targets, and neurobiological consequences is summarized in Table 1.

**Table 1:** Functional classification of bacterial neurotoxins.

Functional Class	Toxin	Bacterial Source	Molecular Mechanism	Primary Neuronal Target	Neurobiological Consequence	Ref
**SNARE-Targeting Neurotoxins**	Botulinum neurotoxins (BoNT/A–G; mosaic variants)	*C. botulinum* *C. baratii*, *C. butyricum*	Zn²⁺- dependent cleavage of SNAP-25, VAMP, syntaxin	Presynaptic SNARE complex	Block of neurotransmitter release	(Rossetto, Pirazzini and Montecucco, 2014; Peck et al., 2017; Pirazzini et al., 2017; Dong, Masuyer and Stenmark, 2019)
	Tetanus neurotoxin (TeNT)	*C. tetani*	VAMP cleavage after retrograde transport	Inhibitory interneurons (CNS)	Spastic paralysis	(Schiavo, Matteoli and Montecucco, 2000; Dong, Masuyer and Stenmark, 2019)
**Pore-Forming Neurotoxins**	Pneumolysin (PLY)	*S. pneumoniae*	Cholesterol-dependent pore formation; Ca²⁺ influx	Neuronal & endothelial membranes	Neuroinflammatio, BBB damage	(Shi et al., 2025)
	Epsilon toxin (ETX)	*C. perfringens* (types B/D)	MAL-dependent oligomeric pore formation	Oligodendrocytes; brain endothelium	Demyelination, excitotoxic injury	(Bokori-Brown et al., 2011; Rumah et al., 2015; Dorca-Arévalo et al., 2024)
**Intracellular Signaling–Modulating Toxins**	TcdA / TcdB	*C. difficile*	Glucosylation of Rho-family GTPases	Rho, Rac, Cdc42	Cytoskeletal collapse	(Just, Wilm, et al., 1995; Voth and Ballard, 2005; Aktories, Schwan and Jank, 2017)
	TcsL / TcsH	*P. sordellii*	Ras/Rho glucosylation	Ras/Rho signaling axis	Disrupted survival pathways	(Genth and Just, 2011; Popoff, 2014)
	C3 exoenzyme	*C. botulinum*	ADP-ribosylation of RhoA	RhoA	Synaptic structural instability	(Barth et al., 1998; Simon, Aktories and Barbieri, 2014)
	Shiga toxin type 2 (Stx2)	*S. dysenteriae* STEC *E. coli*	28S rRNA depurination; ER stress	Gb3-positive neurons	Encephalopathy	(Obata, 2010; Melton-Celsa, 2014; Pinto, Celi and Goldstein, 2023)
**GPCR-Signaling Modulators (Neuroactive)**	Cholera toxin (CT)	*V. cholerae*	ADP-ribosylation of Gsα → ↑cAMP	GM1 ganglioside	Enteric neuronal dysregulation	(Spangler, 1992; Sánchez and Holmgren, 2008)
	Pertussis toxin (PTX)	*B. pertussis*	ADP-ribosylation of Gi/o proteins	GPCR complexes	Altered chemokine & neuronal signaling	(77, 104)

## 3 Neurotoxins and neuroinflammation crosstalk

Bacterial neurotoxins interact directly with glial components of the central nervous system (CNS), establishing bidirectional crosstalk that modulates neuroinflammatory cascades independent of peripheral or microbial triggers (Liddelow et al., 2017). This interplay primarily involves astrocyte reactivity, complement activation, and microglial polarization, leading to either resolution or amplification of neuronal damage (Hammond, Marsh and Stevens, 2019).

BoNT/A exemplifies this duality. At therapeutic single-dose levels, BoNT/A exerts anti-inflammatory effects by targeting glial cells beyond its canonical neuronal SNAP-25 cleavage (Marinelli, 2025). It inhibits NF-κB, p38, and ERK1/2 pathways in microglia and astrocytes, reducing pro-inflammatory mediator release and promoting an anti-inflammatory glial phenotype that supports axonal regeneration and pain modulation in neuropathic models (Feng et al., 2021; Luvisetto, 2022). Recent insights confirm BoNT/A’s capacity to engage both peripheral and central glial networks, offering therapeutic potential in neurological disorders through glial-mediated neuroprotection (Marinelli, 2025).

In contrast, prolonged or repeated exposure to BoNT/A under experimental conditions has been associated with glial activation and secondary neuroinflammatory responses, suggesting context-dependent effects beyond its classical presynaptic SNARE cleavage activity (Wang et al., 2020).

Experimental neuron–astrocyte–microglia co-culture and organoid-based models indicate that BoNT/A can suppress cholinergic neurotransmission through SNAP-25 cleavage and simultaneously modulate glial reactivity, including astrocytic activation and altered secretion of immunomodulatory cytokines such as TGF-β (Luvisetto, 2022).

Reactive astrocytes and stressed neurons can engage complement-associated signaling pathways (e.g., C1q–C3 axis), contributing to microglial recruitment and activation, thereby amplifying neuroinflammatory signaling loops (Liddelow et al., 2017).

Activated microglia can adopt a pro-inflammatory phenotype characterized by increased production of cytokines (e.g., IL-1β, TNF-α) and nitric oxide–associated inflammatory signaling, which is broadly linked to synaptic dysfunction and neuronal vulnerability in experimental neuroinflammation models (Ransohoff, 2016).

These inflammatory cascades have been associated with synaptic pruning, cytoskeletal instability, tau phosphorylation, and neuronal stress responses in diverse neuroinflammatory and neurodegenerative experimental systems (Hong et al., 2016).

The non-toxic C-fragment of tetanus neurotoxin (TTC) provides an additional anti-inflammatory example. In SOD1^G93A^ ALS models, TTC reduces IL-6 in spinal cord and muscle while modulating NLRP3 inflammasome and caspase-1, attenuating glial-driven neurotoxicity without the disinhibitory effects of full-length TeNT (Moreno-Martinez et al., 2020). Overall, these observations are consistent with emerging concepts highlighting neuroimmune communication as an important regulator of immune homeostasis and inflammatory responses (Khatab, Ahmadova and Liju, 2025).

## 4 Neurotoxins and the gut–brain axis

Bacterial neurotoxins produced within the intestinal lumen exploit the microbiota–gut–brain axis (MGBA) to influence CNS homeostasis through neural, humoral, and barrier pathways (Cryan et al., 2019; Morais, Schreiber IV and Mazmanian, 2021). Unlike direct glial crosstalk, this route begins with luminal colonization, protease activation, and systemic dissemination, enabling distant neurotoxic effects without initial CNS entry (Uzal et al., 2014).

Epsilon toxin (ETX) from *Clostridium perfringens* types B and D represents the most well-characterized example of a microbiota-derived bacterial neurotoxin implicated in gut–brain axis communication. ETX is produced as an inactive protoxin within the intestinal lumen and becomes activated following proteolytic processing by host enzymes such as trypsin and chymotrypsin (Fernandez Miyakawa and Uzal, 2003; Stiles et al., 2013). After activation, the toxin can translocate across the intestinal epithelium and enter systemic circulation, providing a mechanistic route for peripheral exposure to a CNS-targeting bacterial toxin. This gut-derived dissemination pathway highlights the capacity of microbial products to reach distant neural compartments through barrier-mediated transport systems, without requiring direct CNS colonization (Linden et al., 2015).

Pioneering human microbiome analyses further quantified this association: *etx* prevalence reached 61% in MS patients versus 13% in age- and sex-matched healthy controls (odds ratio 10.7, *P* = 0.0002), with ETX-producing strains comprising 32–43% of total *C. perfringens* in MS feces versus ∼0.001% in controls. In experimental autoimmune encephalomyelitis (EAE) models, ETX substitution for pertussis toxin induced multifocal BBB disruption, leukocyte infiltration, perivascular cuffing, and demyelinating lesions distributed across the corpus callosum, thalamus, cerebellum, brainstem, and spinal cord, patterns mirroring human MS topography. These effects occur via gut-derived ETX overcoming CNS immune privilege, with episodic *C. perfringens* blooms in dysbiotic microbiomes providing the luminal source (Dorca-Arévalo et al., 2024).

Broader MGBA reviews emphasize that clostridial taxa, including *Clostridium* species, contribute to axis dysregulation through metabolite and toxin profiles that alter vagal signaling and immune trafficking, though ETX remains the primary pore-forming exemplar. Therapeutic strategies targeting MGBA therefore include microbiome modulation to suppress ETX-producing colonization or neutralizing antibodies to prevent systemic transit (Fung, Olson and Hsiao, 2017; Loh et al., 2024; Faysal et al., 2025).

Although these observations strengthen the proposed involvement of bacterial neurotoxins in gut–brain communication, toxin production and host susceptibility are shaped by a complex interplay between microbial ecology and host biology. Differences in microbiome composition and environmental influences may partially account for the variability reported among experimental and clinical studies (Cryan et al., 2019; Sorboni et al., 2022). Recent evidence further suggests that gut microbiota-derived neurotransmitters contribute to neuroimmune regulation and may influence immune homeostasis across neurological disorders, underscoring the broader complexity of microbiota–brain communication beyond microbial toxin production (Marra, Liju and Ghaffar, 2025).

Therefore, further investigation in independent experimental settings and carefully characterized patient cohorts will be important for refining these associations and defining their translational relevance.

## 5 Neurotoxins and neurodegenerative disorders

Bacterial neurotoxins contribute to core neurodegenerative processes-protein aggregation, selective neuronal vulnerability, and progressive synaptic/axonal degeneration-through mechanisms distinct from acute inflammation or gut-origin dissemination (Kampmann, 2024; Koszła and Sołek, 2024).

In Parkinson’s disease (PD), BoNT/A has established symptomatic efficacy for dystonia, sialorrhea, and tremor via peripheral neuromuscular blockade (Grippe and Chen, 2023). Systematic reviews confirm improved motor function and quality of life with targeted injections (Safarpour and Jabbari, 2018; Jocson and Lew, 2019). Emerging protein-engineering approaches exploit BoNT protease scaffolds to selectively degrade disease-associated proteins: reprogrammed BoNT-derived proteases have been shown to clear α-synuclein aggregates in human iPSC-derived dopaminergic neurons and rodent PD models, restoring lysosomal function and mitigating dopaminergic loss without native toxicity (Sondermann et al., 2025). This repurposing transforms a classical neurotoxin into a precision tool against intraneuronal proteinopathy.

In multiple sclerosis (MS), viewed as a chronic neurodegenerative condition with progressive axonal loss, ETX directly targets oligodendrocytes via MAL receptors, inducing selective cell death, myelin decompaction, and multifocal axonal degeneration in preclinical models (Linden et al., 2015; Ma et al., 2023). Ultrastructural analyses reveal splitting and vacuolization of myelin sheaths, independent of peripheral immune priming, contributing to cumulative disability (Finnie and Uzal, 2022).

For amyotrophic lateral sclerosis (ALS), the non-toxic tetanus toxin C-fragment (TTC) demonstrates direct neuroprotective effects on motor neurons (Bayart et al., 2022). In SOD1^G93A^ transgenic mice, TTC administration preserved neuromuscular junctions, reduced motor neuron apoptosis, and extended survival by modulating intracellular trafficking and anti-apoptotic pathways, offering a non-inflammatory therapeutic scaffold (Patricio et al., 2019; Zhao et al., 2019).

These disease-specific actions underscore the potential of bacterial neurotoxins and their derivatives as both pathological contributors and engineered biologics in neurodegenerative proteinopathies and motor neuron diseases, with ongoing translational efforts focused on selectivity and delivery (Webb, 2018).

Although accumulating evidence supports potential associations between BoNT, epsilon toxin, and, to a lesser extent, Clostridioides difficile toxins with neuroinflammatory and neurodegenerative processes, comparable evidence remains limited for several other bacterial neurotoxins (Popoff and Poulain, 2010; Catumbela et al., 2023; Zhang et al., 2023). For example, pneumolysin has been primarily investigated in the context of acute pneumococcal meningitis rather than chronic neurodegeneration (Yang et al., 2023). In contrast, tetanus neurotoxin has been extensively used as an experimental tool for neuronal tracing and circuit mapping rather than being implicated as a causative factor in progressive neurodegenerative diseases (Skryabina et al., 2023). Similarly, the neurological effects of cholera toxin and pertussis toxin are mainly related to intracellular signaling modulation and experimental neurobiology, with little evidence supporting a direct role in chronic neurodegenerative disorders (Locht, 2023; Miguelena Chamorro et al., 2023). Therefore, current knowledge is uneven across toxin families, and the proposed links between bacterial neurotoxins and chronic neurodegeneration should be interpreted cautiously until supported by broader mechanistic and clinical evidence.

## 6 Research and therapeutic applications of bacterial neurotoxins

Bacterial neurotoxins can be defined as molecular modulators of neural function that have evolved to interact with critical regulatory nodes of neuronal signaling networks, rather than solely acting as cytotoxic agents (Bagues et al., 2024). This molecular precision is responsible for their pathogenic potency and translational potential (Rasetti-Escargueil and Palea, 2024).

One of the most well-established therapeutic applications is the clinical use of purified derivatives of botulinum neurotoxins produced by *Clostridium botulinum* (Zhantleuova et al., 2024). BoNT/A formulations are currently the first-line therapies for post-stroke spasticity, focal dystonia, blepharospasm, and chronic migraine (Dodick et al., 2010; Simpson et al., 2016; Francisco et al., 2020; Suputtitada et al., 2024). Large multicenter randomized clinical trials have shown significant reductions in muscle tone and improved functional outcomes with minimal systemic toxicity when administered locally and at controlled doses (Otero-Luis et al., 2024).

In addition to motor disorders, controlled clinical studies have demonstrated sustained analgesic effects of BoNT/A in peripheral neuropathic pain. These effects involve suppression of nociceptive neuropeptides such as CGRP and substance P in sensory terminals, beyond acetylcholine inhibition, as confirmed by electrophysiological and biochemical analyses (Bagues et al., 2024). These findings support the concept that neurotoxins can selectively modulate distinct neurotransmitter and neuropeptide pools (Rasetti-Escargueil and Palea, 2024).

In research settings, different BoNT serotypes that target discrete SNARE components have become essential tools for dissecting vesicular fusion dynamics. In research settings, BoNTs serve as precision molecular tools for the selective and reversible blockade of synaptic transmission, allowing controlled interrogation of neurotransmitter release dynamics and circuit-level functional organization in neuronal systems (Pirazzini et al., 2017).

LCTs produced by *Clostridioides difficile* have also been used as experimental tools for selectively inactivating RhoA, Rac1, and Cdc42 in neuronal models. Controlled in vitro applications have helped clarify the role of Rho GTPases in dendritic spine remodeling, actin cytoskeleton organization, and synaptic stability (Aktories, Schwan and Jank, 2017).

Advances in protein engineering have expanded translational opportunities. Recombinant BoNT variants with reduced catalytic activity but intact neuronal binding domains are being studied as targeted delivery vectors. Preclinical studies show that linking these targeting domains to therapeutic cargos enables selective delivery to cholinergic neurons, demonstrating their potential as neuron-specific transport platforms (Zhantleuova et al., 2024).

Concurrently, research on pore-forming toxins has investigated cholesterol-mimetic molecules that can block pneumolysin-induced membrane damage in experimental meningitis models (Sgrulletti et al., 2025). These findings suggest the possibility of designing toxin-neutralizing strategies.

These advances collectively support the expanding role of engineered neurotoxins in translational neurology and precision neuromodulation (Rasetti-Escargueil and Palea, 2024; Zhantleuova et al., 2024).

## 7 Biotechnological engineering of bacterial neurotoxins

### 7.1 Recombinant and detoxified neurotoxin derivatives

Recently, engineered and catalytically inactive BoNTs have been identified as valuable biological platforms for the delivery of biological cargos into neuronal cells. In such engineered toxins, while the BoNT receptor binding and translocation domains remain intact, their metalloprotease activity, which requires the presence of Zn²⁺ ions, is ablated to limit toxic effects. Research on BoNT binding and translocation domains has revealed that they possess neuronal cell-targeting capabilities when integrated into engineered constructs (Dressler and Johnson, 2022; Lalaurie et al., 2022). Furthermore, the development of engineered BoNT/A derivatives has suggested that rational structural modification may improve neuronal uptake efficiency and intracellular cargo delivery in experimental neuronal systems (Wei et al., 2022).

Apart from being used as neuronal carriers for drugs, the BoNT recombinant derivatives have been used in vaccine formulation research. It has been reported that recent studies on the development of recombinant vaccines to target BoNT/A and BoNT/B toxins resulted in the ability to create BoNT derivatives that produce a neutralizing antibody response, but not neurotoxicity due to rational structural modifications (Ayoub, 2025). Overall, these reports show that rational bioengineering of bacterial neurotoxins has come a long way from detoxification experiments and reached the level of translation for use in the field of neuroscience and neurotherapy.

In line with detoxified neurotoxin engineering approaches, atoxic BoNT/C1 ad toxin was created by modifying the metalloprotease domain by rational amino acid replacements in order to preserve the property to target neurons. Experiments revealed that the recombinant protein retains its high affinity to internalize presynaptically into neuronal membranes without causing SNARE cleavage and neurotransmission disruption. In addition, in vivo experiments revealed that an atoxic derivative of BoNT/C1 (BoNT/C1 ad) selectively localized to motor nerve terminals in the mouse diaphragm without exhibiting systemic toxicity compared with the wild-type toxin (Vazquez-Cintron et al., 2017).

### 7.2 Engineered neurotoxins for circuit-specific neuromodulation

BoNT/A neurophysiological effects at the level of neural networks have also been supported by recent brain imaging investigations. In a functional magnetic resonance imaging study performed by Vinehout et al., patients treated with BoNT/A for post-stroke spasticity showed different patterns of cortical activity and changes in functional connectivity of the sensorimotor networks. This implies a possible role of the drug as an inducer of neuroplasticity, apart from its already known peripheral inhibitory actions (Vinehout et al., 2021).

### 7.3 Synthetic biology and precision engineering of neurotoxins

Advances in synthetic biology and protein engineering have expanded the potential of bacterial neurotoxins as programmable experimental platforms for targeted neural modulation. Structural studies have shown that distinct functional domains of BoNTs can be redesigned to alter receptor binding, cellular entry, and neuronal selectivity. Such approaches include receptor-binding domain engineering, modification of cellular tropism, and the development of chimeric toxin architectures with altered functional properties (Lalaurie et al., 2022; Jahromi et al., 2025).

Contemporary studies on engineered neurotoxins further indicate that structure-guided rational design strategies can improve functional selectivity and biological stability in experimental systems. In addition, the integration of computational modeling and advanced protein engineering methods is supporting the development of less toxic and more precisely targeted neurotoxin variants (146). Collectively, these advances suggest that bacterial neurotoxins are being increasingly investigated as platforms for precision neuroengineering and translational neuromodulation.

Recent advances in structure-guided protein engineering have enabled the development of botulinum neurotoxin variants with enhanced receptor recognition and targeting specificity. For example, contemporary studies have reported the structure-based engineering of recombinant BoNT variants capable of interacting with multiple neuronal receptor systems simultaneously through rational modification of receptor-binding domains. These engineered neurotoxins demonstrated altered receptor recognition properties and improved neuronal targeting potential, highlighting the growing role of synthetic biology and structural bioengineering in the development of precision neurotoxin platforms for translational neurotherapeutics (Masuyer and Stenmark, 2024).

Despite these advances, several practical issues continue to influence the development of engineered bacterial neurotoxins for biomedical applications. The production of recombinant neurotoxin derivatives requires preservation of structural stability and functional reproducibility while maintaining an acceptable safety profile throughout manufacturing. In addition, successful clinical translation will depend on comprehensive preclinical evaluation, quality assurance during production, and compliance with regulatory standards governing biologically engineered therapeutics (Brin et al., 2024; Zhantleuova et al., 2024). Addressing these aspects alongside continued advances in protein engineering will be essential for translating experimental neurotoxin platforms into clinically applicable technologies.

## 8 Challenges and limitations in the clinical exploitation of bacterial neurotoxins

Despite the significant therapeutic potential of bacterial neurotoxins, particularly BoNT/A, in treating spasticity, movement disorders, chronic pain, and selected neurological conditions, their broader clinical use is limited by immunogenicity, safety concerns, regulatory barriers, and unresolved translational challenges (Brideau-Andersen, Dolly and Brin, 2023; Rasetti-Escargueil and Palea, 2024). Experimental applications involving tetanus toxin fragment C (TTC) and epsilon toxin (ETX) mechanisms also face limitations that preclude clinical implementation.

One major limitation of long-term BoNT/A therapy is the development of neutralizing antibodies (NAbs), which can lead to secondary treatment failure (STF) or reduced therapeutic effectiveness over time. The risk of immunogenicity increases with high cumulative doses, frequent reinjection intervals, and formulations containing complexing proteins. While newer purified preparations like incobotulinumtoxinA have lower immunogenicity rates, some chronically treated patients may still develop antibody-mediated resistance, requiring dose adjustments, formulation changes, or temporary treatment pauses (Bellows and Jankovic, 2019). Similar concerns may apply to engineered neurotoxin derivatives or TTC-based delivery constructs for chronic neurodegenerative disorders.

Current strategies to mitigate immunogenicity in engineered bacterial neurotoxins rely on rational protein engineering, including epitope modification and the development of alternative serotype-based or chimeric variants intended to preserve biologic activity while reducing antigen recognition (Grinberg and Benhar, 2017; Harris and Cohen, 2024). Nevertheless, the long-term effects of repeated exposure to these engineered derivatives remain insufficiently defined, and prolonged clinical follow-up will be required to establish sustained efficacy, immunogenicity profiles, and safety (Liu et al., 2024).

Safety is another important consideration. Local diffusion of BoNT/A beyond injection sites can cause unintended muscle weakness, dysphagia, respiratory issues, or systemic botulism-like symptoms, especially in vulnerable patients with high-dose treatments (Pirazzini et al., 2017). Counterfeit or mishandled toxin preparations raise concerns about manufacturing oversight, supply chain integrity, and administrative standards (Wang et al., 2025). Potential long-term neuroimmune consequences of repeated toxin exposure remain insufficiently characterized clinically.

Translational research on ETX faces even greater challenges. While ETX-producing Clostridium perfringens strains are more common in some MS and NMOSD patient groups, the evidence is mostly associative rather than definitively causal (Bijjam et al., 2024). Variability exists in toxin detection, microbial abundance thresholds, and reproducibility across populations. Given ETX's pore-forming and blood-brain barrier-disrupting properties, any therapeutic strategies would need precise targeting to avoid potential demyelination or neurovascular damage.

Production and regulatory barriers further complicate matters. Potency assessment for some neurotoxin formulations still relies on animal-based bioassays, like murine lethality testing, despite efforts to develop cell-based alternatives for improved reproducibility and ethical concerns.

(Bellows and Jankovic, 2019; Dressler and Frevert, 2026).

High manufacturing costs, cold-chain storage requirements, limited accessibility in resource-constrained settings, and the proliferation of biosimilar or counterfeit preparations further complicate equitable clinical implementation (Carr, Jain and Sublett, 2021; Martin, Frevert and Tay, 2024).

Translational limitations also remain substantial for emerging bioengineered applications. Recombinant BoNT scaffolds and TTC-derived neuronal targeting systems have shown promising experimental utility as selective delivery platforms for therapeutic cargos. However, major obstacles persist, including optimization of CNS delivery, translation of preclinical findings, heterogeneity of patient responses, and the absence of validated biomarkers for monitoring efficacy and toxicity (Pirazzini et al., 2017; Dong, Masuyer and Stenmark, 2019; Dressler and Johnson, 2022). Furthermore, although BoNT/A provides well-established symptomatic improvement in disorders such as dystonia and post-stroke spasticity, current evidence does not support a definitive disease-modifying effect in progressive neurodegenerative diseases (Wang et al., 2025).

Collectively, available evidence indicates that bacterial neurotoxins represent highly promising yet biologically complex therapeutic platforms. Despite these advances, the current body of evidence remains constrained by several important limitations. Much of the mechanistic understanding is derived from experimental models, whereas longitudinal clinical studies and large multicenter investigations remain limited. Furthermore, variability in toxin exposure, host susceptibility, experimental methodologies, and patient populations complicates direct comparisons across studies (Yoelin and Hooper, 2024). Future research should therefore emphasize standardized experimental approaches, well-characterized clinical cohorts, and multidisciplinary integration of structural biology, microbiome science, and protein engineering to facilitate the safe translation of bacterial neurotoxins into precision neurological therapies. Continued progress in structural engineering, targeted delivery technologies, immunogenicity reduction, and long-term safety characterization will be essential for expanding their future role in precision neurology and translational neuroscience (Fonfria et al., 2018; Rasetti-Escargueil and Palea, 2024).

## 9 Conclusions

Bacterial neurotoxins are highly specialized modulators of neuronal function that have evolved remarkable molecular precision in targeting synaptic transmission, membrane integrity, and intracellular signaling pathways. Through mechanisms such as SNARE-directed proteolysis, pore-mediated membrane permeabilization, and inactivation of small GTPases, these toxins disrupt neuronal homeostasis at multiple structural and functional levels.

Beyond their acute neurotoxic effects, bacterial neurotoxins are increasingly implicated in neuroinflammatory signaling, gut–brain axis dysregulation, and chronic neurodegenerative processes. Their capacity to modulate glial activation, synaptic stability, protein homeostasis, and neuronal survival suggests a broader role in central nervous system pathology than previously appreciated. At the same time, advances in structural biology, protein engineering, and neurobiological modeling have transformed these molecules into valuable experimental and therapeutic platforms. Engineered neurotoxin derivatives and toxin-based delivery systems are increasingly being explored for targeted neuromodulation, neuroprotection, and precision therapeutic applications in disorders such as Parkinson’s disease, amyotrophic lateral sclerosis, multiple sclerosis, and chronic pain.

Despite these advances, significant translational challenges remain, including immunogenicity, tissue specificity, long-term safety, and optimization of CNS-targeted delivery. Further integration of molecular neuroscience, microbiome research, and bioengineering approaches will be essential to refine the therapeutic potential of these biologically complex molecules. Collectively, bacterial neurotoxins are emerging not only as mediators of neuropathology but also as powerful tools for understanding and selectively manipulating neural systems in translational neuroscience.

## CRediT authorship contribution statement

**Hanieh Banaeyoun:** Conceptualization, Methodology, Writing – original draft, Software. **Yaser Solhi Digehsara:** Data curation, Writing – original draft. **Behrouz Golichenari:** Supervision, Investigation, Visualization, Writing – review & editing.

## Declaration of competing interest

The authors declare that they have no known competing financial interests or personal relationships that could have appeared to influence the work reported in this paper.
